# Sclerodermatous Graft-Versus-Host Disease: A Rare Sequela of Hematopoietic Stem Cell Transplantation

**DOI:** 10.7759/cureus.47963

**Published:** 2023-10-30

**Authors:** Mohamad Jabin, Sonali Batta, Madeeha Mian, Palak Parekh

**Affiliations:** 1 Medicine, Texas A&M University School of Medicine, Bryan, USA; 2 Dermatology, Baylor Scott and White Medical Center - Temple, Temple, USA

**Keywords:** eosinophilic infiltration, acute myeloid leukemia, sclerodermatous, hematopoetic stem cell, graft versus host

## Abstract

Although graft-versus-host disease (GVHD) is a common immunologic reaction after an allogeneic hematopoietic stem cell transplant (HSCT), progression into sclerodermatous GVHD is a rare sequela. It can present locally or generalized with various cutaneous and mucocutaneous manifestations, resulting in loss of skin elasticity and reduced functional capabilities. One of the most debilitating consequences of sclerodermatous GVHD is its effect on a range of motion due to fibrosis of the skin and subcutaneous fat. We present the case of a 54-year-old male with a medical history of acute myeloid leukemia and an allogeneic stem cell transplant who was diagnosed with sclerodermatous GVHD. We review the characteristic clinical and histopathological findings of sclerodermatous GVHD, as well as its treatment. Early recognition and intervention are crucial to prevent complications, such as joint contractures.

## Introduction

Graft-versus-host disease (GVHD) is an adverse immunologic reaction that can take shape after an allogeneic hematopoietic stem cell transplant (HSCT) [1]. This reaction occurs when donor T-cells respond to host cell proteins, notably human leukocyte antigens (HLAs) [2]. GVHD affects roughly 40% to 60% of patients who undergo HSCT, with a mortality rate of 15% [3]. GVHD is further subdivided into acute and chronic. Acute GVHD develops within 100 days, while chronic GVHD develops after 100 days. A rare sequela of GVHD is the progression to sclerodermatous GVHD, which occurs in 3% of patients who undergo HSCT [4]. Sclerodermatous GVHD can present locally or be generalized and is characterized by features such as atrophy, sclerosis, telangiectasias, hypo- or hyperpigmentation, contractures, hair loss, ulcerations, and koilonychia of the nails. It may also display mucocutaneous findings, including xerostomia and sclerodermatous plaques involving the oral cavity and lips [5].

## Case presentation

**Figure 1:**
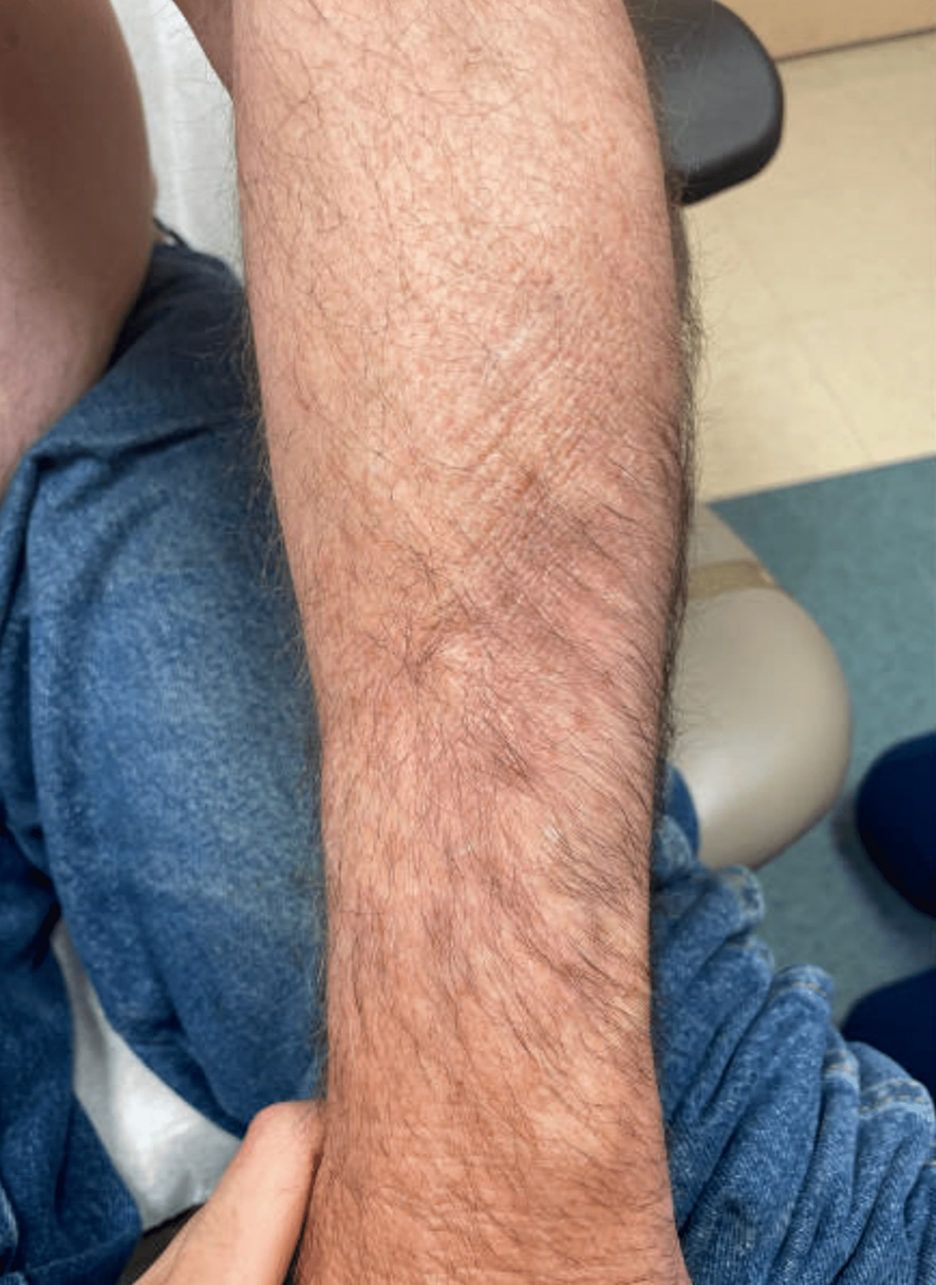
Hypertrophic and hyperpigmented lesions on the patient's left upper extremity.

**Figure 2:**
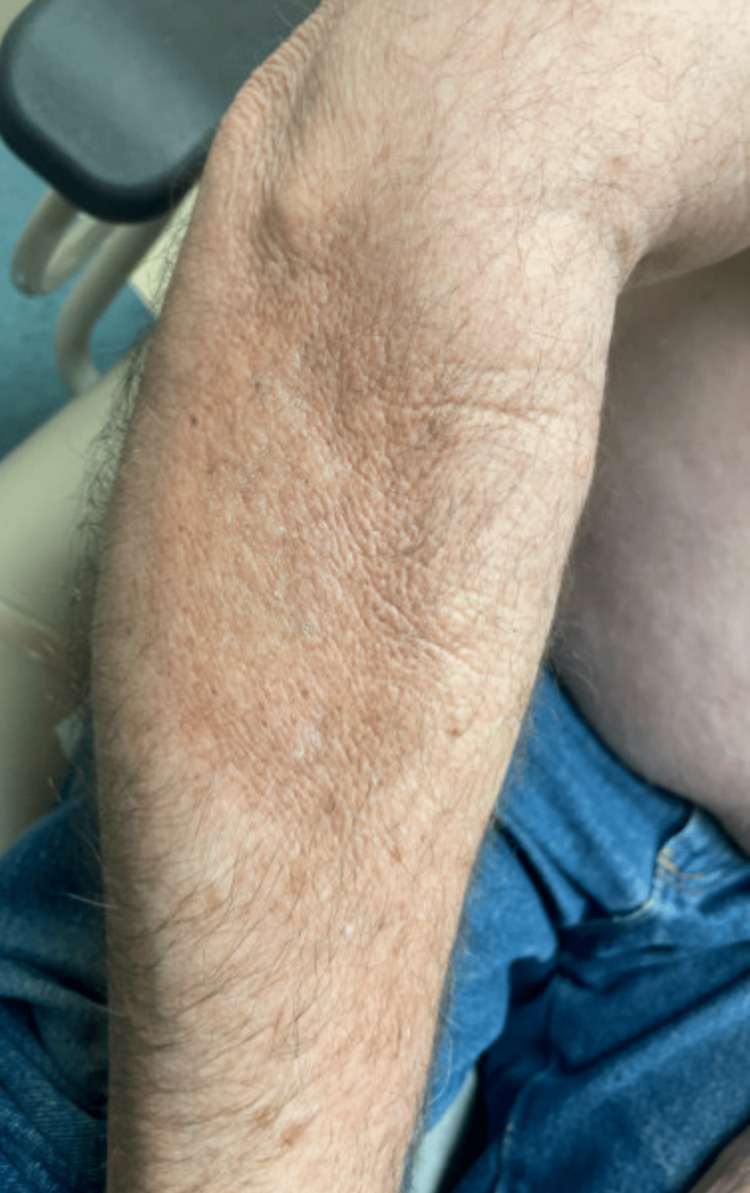
Hypertrophic and hyperpigmented lesions on the patient's right upper extremity.

**Figure 3:**
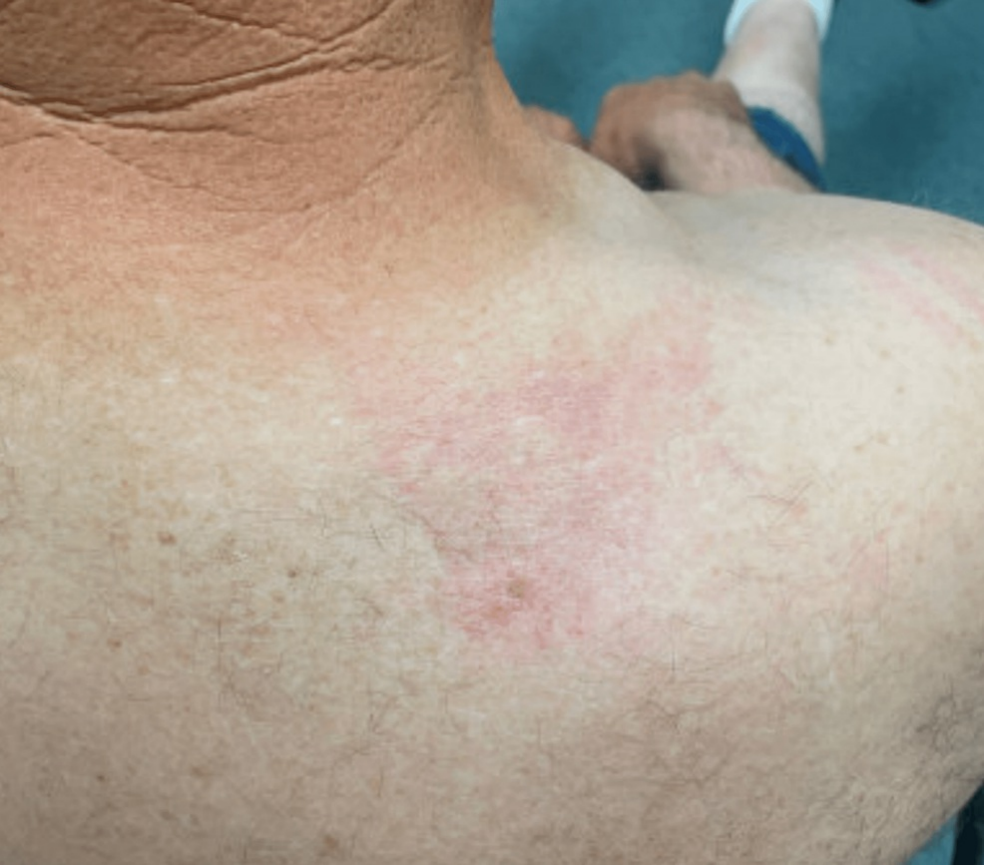
Early sclerodermatous changes showing an erythematous lesion with telangiectasias on the patient's posterior right shoulder.

A 54-year-old male with a history of non-melanoma skin cancer, acute myeloid leukemia (AML), and allogeneic stem cell transplant (2015) presented as an outpatient for a routine skin check due to his history of being immunocompromised. During his evaluation, he noted a dry scaly lesion on his right lateral elbow. The patient stated that he had a blood clot in his right arm a couple of years ago and the texture of his skin has not been the same since. He also admitted to the lesion becoming increasingly scaly over the past few months. On exam, an indurated plaque with dilated pores and peau d’orange appearance was observed on the patient’s bilateral upper extremities (Figures 1-2). Upon further inspection, it was also noted that the patient had an erythematous lesion with telangiectasias on his posterior right shoulder (Figure 3). Due to the suspicious findings and the patient's history of bone marrow transplant, a 4-mm punch biopsy was performed on his right forearm.

The biopsy revealed superficial and deep lymphoplasmacytic inflammation, scattered eosinophils, and deep dermal sclerosis extending to the base of the specimen. Complete blood count with differential was within normal limits and notably negative for eosinophilia. A complete metabolic panel indicated a slightly elevated creatinine but showed no other abnormalities. Erythrocyte sedimentation rate, serum protein electrophoresis, and antinuclear antibody returned negative. The patient declined the fascial biopsy and elected to treat the lesion empirically with clobetasol ointment. Although the punch biopsy was superficial to fascia, the histological findings in combination with the characteristic clinical features of induration and peau d’orange textural changes suggested a diagnosis of sclerodermatous GVHD. Given his history of AML, the hematology/oncology service was consulted, and the diagnosis was subsequently confirmed. The patient was then placed on clobetasol cream to use twice a day on the affected areas for the next month and then taper down to triamcinolone cream. Unfortunately, we were unable to monitor the disease's progression as the patient succumbed to sudden cardiac death two months later.

## Discussion

It is important to note that localized sclerodermatous GVHD can occur as an isolated finding, without progression to chronic widespread GVHD. However, generalized sclerodermatous GVHD, with its more extensive skin involvement, is only seen in patients who have already developed chronic GVHD. One of the most debilitating consequences of this disease is its effect on range of motion (ROM). Fibrosis of the skin and subcutaneous fat results in loss of skin elasticity, thus reducing ROM, strength, endurance, and functional capabilities of the affected site. Characteristic features on skin biopsy of sclerodermatous GVHD include an atrophic or normal epidermis, vacuolar degeneration of the basal cell layer, inflammation with eosinophilic infiltration, pigmentary incontinence in the dermis, and destruction of adnexal structures in the dermis with fibrosis, possibly accompanied by fibrosis extending to the subcutaneous fat [4]. The mainstay of treatment of sclerodermatous GVHD is topical/oral corticosteroid use and phototherapy. Recent studies have also shown that the use of calcineurin inhibitors like tacrolimus and pimecrolimus have potential benefits in reducing erythema and pruritus in up to 72% of patients who have steroid-refectory sclerodermatous GVHD [6]. It has also been reported that the use of phototherapy alongside immunosuppressive therapy has proven to be a successful form of treatment [7]. Budesonide mouthwash has shown significant efficacy for oral mucosa involvement.

## Conclusions

The progression to sclerodermatous GVHD following an allogeneic HSCT presents a rare but significant challenge. This condition, whether localized or generalized, leads to a range of cutaneous and mucocutaneous manifestations, often culminating in compromised skin elasticity and reduced functional capacity. Of particular concern is its impact on ROM due to skin and subcutaneous fat fibrosis. The case presented here underscores the need for early recognition and treatment to mitigate complications like joint contractures. With the prevalence of GVHD in transplant recipients, understanding the characteristic clinical and histopathological markers of sclerodermatous GVHD remains paramount. Treatment options, including corticosteroids, phototherapy, and emerging immunosuppressive agents, have demonstrated efficacy, underscoring the importance of prompt intervention. As our understanding of this condition deepens, a collaborative effort among clinicians, hematologists, and dermatologists remains essential to optimize management strategies and enhance patients' quality of life.
